# Effectiveness comparisons of acupuncture for chronic prostatitis/chronic pelvic pain syndrome: A Bayesian network meta-analysis protocol

**DOI:** 10.1097/MD.0000000000015199

**Published:** 2019-04-26

**Authors:** Yi Lei, Xueyun He, Jingshang Wang, Xiaoyong Gong, Wei Zheng, Yahui Xue, Yongqiang Li, Bao Zhang, Jiajia Ma, Chaohui Xue

**Affiliations:** a The Second Affiliated Hospital of Shaanxi University of Traditional Chinese Medicine; b Shangluo Central Hospital in Shaanxi Province; c Department of Traditional Chinese Medicine, Beijing Obstetrics and Gynecology Hospital, Capital Medical University, Chaoyang District; d Dongzhimen Hospital, Beijing University of Chinese Medicine; e Beijing Airport Hospital, Shunyi District, Beijing, China.

**Keywords:** acupuncture, chronic pelvic pain syndrome, chronic prostatitis, systematic review

## Abstract

**Background:**

Chronic prostatitis/chronic pelvic pain syndrome (CP/CPPS) is a common urinary system disease in the male population. Recent studies have shown that acupuncture can alleviate the pain caused by CP/CPPS to a certain extent and improve the quality of life of patients. This study used a network meta-analysis (NMA) to compare the effectiveness and safety of different forms of acupuncture on CP/CPPS.

**Methods and analysis:**

We will search for PubMed, Cochrane Library, AMED, EMbase, WorldSciNet; Nature, Science online and China Journal Full-text Database, China Biomedical Literature CD-ROM Database, and related randomized controlled trials (RCTs) included in the China Resources Database. The time is limited from the construction of the library to December 2018. The quality of the included RCTs will be evaluated with the risk of bias tool and evidence will be evaluated by grading of recommendations assessment, development, and evaluation. STATA 13.0 and WinBUGS 1.4.3 through the GeMTC package will be used to perform a NMA to synthesize direct and indirect evidence.

**Results:**

The results of this NMA will be submitted to a peer-reviewed journal for publication.

**Trial registration number:**

PROSPERO CRD42018111408.

## 1 Introduction

Chronic prostatitis/chronic pelvic pain syndrome (CP/CPPS), a common urinary system disease in the male population, has the symptoms of repeated, long-term pain and discomfortableness around pelvic floor area and lumbosacral portion, besides, varying degrees of lower urinary tract symptoms (frequent urination, urgency, dysuria, urinary incontinence, etc) would also become visible.^[1–2]^ Some patients may also suffer from dizziness, memory loss, sexual dysfunction, and even depression.^[3]^ Epidemiological investigations have shown that 2.2% to 13.8% of adult men are suffering from it, and approximately 30% to 50% of men would suffer from it in particular periods of their lives. Related literature reports that the prevalence of CP/CPPS and the related symptoms of it in China is as high as 46.6%.^[4–5]^ Its impacts not only deteriorate the patients‘ physical and psychological health, but also the social economy, which, because of the high incidence and the recurrence. Thus, the National Institutes of Health (NIH) has listed CP as one of the chronic diseases that affect the quality of life of residents, which include myocardial infarction, unstable angina, and active Crohn disease.^[6]^

The pathogenesis of CP/CPPS is complicated which has not yet been completely illuminated. Literature reports that there are kind of connections between prostate hyperplasia and CP/CPPS.^[7–8]^ At present, there is no specific therapy for this disease in modern medicine and we mainly adopt antibiotics, α-blockers, and so on; however, multicenter clinical studies have shown that the curative effect of single treatment mode is limited or even invalid.^[9–11]^ In addition, due to the specificity of the anatomical structure of prostate – the deeper intima of the capsule, it is easy to cause local microcirculation disturbance, blockage of blood stasis, drainage blocked. All the factors above make the drug difficult to reach the lesion and cannot fully effective, which make the disease become recurrent and chronic.^[12]^

Acupuncture, 1 essential part of traditional Chinese medicine (TCM), has been widely used in clinical trials recently. Recent studies have shown that there are remarkable effects in reducing chronic pain and tissue fibrosis around the pelvic floor area.^[13–15]^ Studies also show that acupuncture could accelerate the central nervous system to produce endogenous opioid peptides and activate relevant receptors by stimulating related acupoints, which achieves peripheral analgesia. Besides, it could also achieve the anti-inflammatory effects by promoting the levels of β-Ep in inflammatory tissue and serum.^[16–18]^ TCM believes that acupuncture can regulate the balance of qi and blood in the human body and the function of body could also be improved by stimulating acupoints. Moreover, it becomes increasingly popular because of its unique advantages of simplicity, convenience, efficacy, and inexpensive.

After preliminary search and analysis of the database, we found that the frequency of randomized controlled trials (RCTs) of acupuncture treatment in CP/CPPS has been showing an increasing trend.^[19]^ Previous clinical trials have shown that acupuncture could ameliorate pain and improve the quality of lives in patients who suffer from CP/CPPS, and these effects are sustained.^[17,20]^ However, due to the limitation of the scale and sample size of the clinical centers, the current level of evidence-based medical evidence is still not sufficient. Therefore, we hope to evaluate the efficacy and safety of acupuncture in treating CP/CPPS by using network meta-analysis, which aims to provide sufficient evidence for its clinical application.

## 2 Methods

This systematic review protocol has been registered on PROSPERO as CRD4218111408 (http://www.crd.york.ac.uk/PROSPERO/display_record.php?ID=CRD42018111408). The protocol follows the Cochrane Handbook for Systematic Reviews of Interventions and the Preferred Reporting Items for Systematic Reviews and Meta-Analysis Protocol (PRISMA-P) statement guidelines. We will describe the changes in our full review if needed.

### 2.1 Inclusion criteria

#### 2.1.1 Types of studies

This study will include all the RCTs that relate to acupuncture therapy in treating CP/CPPS. For the included trials, the investigators need to precisely report the stochastic methods, acupuncture treatment details and parameters, diagnostic criteria and efficacy evaluation they based on. No limitation to whether it is published or not. The experiment is limited to humans. Language is limited to Chinese and English.

#### 2.1.2 Types of participants

Male patients who were definitely diagnosed with CP/CPPS would be included (refer to the NIH diagnostic criteria for CP/CPPS expert consultation). The cases which relate to prostatic hyperplasia, prostate cancer, or other prostate-related diseases would be excluded. In addition, there is no limitation in region, citizenship, nationality, and source of cases.

#### 2.1.3 Types of interventions

##### 2.1.3.1 Experimental interventions

The intervention will include all piercing acupuncture, including hand-acupuncture, electroacupuncture, fire needle, plum blossom needle, abdominal needle, and so on. Other nonpiercing acupunctures such as acupressure, acupressure, acupressure, moxibustion, and so on will be excluded. Acupoint injections will also be excluded. Pharmaco-acupuncture-true and acupoint injection will be rejected, we consider that their methods and theories are different from TCM. The treatment duration and frequency are not limited.

##### 2.1.3.2 Control interventions

The control interventions will include a placebo, a virtual acupuncture, a conventional drug such as an alpha-blocker, an antibiotic, a botanical preparation, a nonsoul anti-inflammatory drug, and the like. However, RCT of acupuncture combined with drugs or other Chinese medicine methods will be excluded.

#### 2.1.4 Types of outcome measures

##### 2.1.4.1 Primary outcomes

The National Institutes of Health's Symptom Score Index (NIH-CPSI) score for CP/CPPS will be used as the primary outcome measure. Every dimension (degree of pain, urinary symptoms, and quality of life) of NIH-CPSI will be evaluated, and the lower the total score, the lighter the clinical symptoms of the patient.

##### 2.1.4.2 Secondary outcomes

Secondary outcomes include:

(1) changes in international prostate symptom scores before and after treatment;
(2) changes in international erectile function scores-5 before and after treatment;
(3) comparison of effective rates between groups and the incidence of adverse events.

### 2.2 Data source.

Database Search: PubMed, Cochrane Library, AMED, EMbase, World SciNet, Nature, Science online and China National Knowledge Infrastructure, China Biomedical Literature CD-ROM Database, China Resources Database. Search for clinical research literature on acupuncture CP/CPPS published in domestic and foreign biomedical journals from the establishment of the library to December 2018. Based on the standards of the Cochrane Collaboration Workbook of the International Evidence-Based Medicine Center, a manual and computer-based method will be used to conduct related literature searches. The search terms include: chronic prostatitis, chronic pelvic floor pain syndrome, nonbacterial prostatitis, acupuncture, acupuncture therapy, acupuncture, electroacupuncture, fire needle, plum blossom needle, skin needle, and abdominal needle. Manually search for topics, abstracts, and so on related to the research of Chinese Journal of Male Science, Chinese Acupuncture and Acupuncture.

### 2.3 Data collection and analysis

#### 2.3.1 Selection of studies

**Figure 1:**
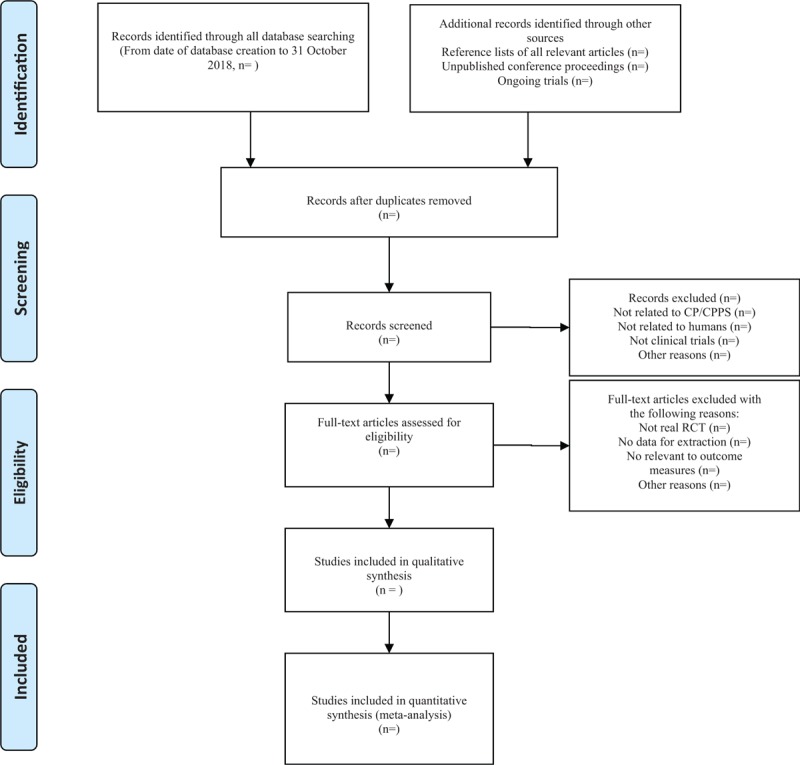
From: Moher D, Liberati A, Tetzlaff J, Altman DG, The PRISMA Group (2009). Preferred Reporting Items for Systematic Reviews and Meta-Analyses: The PRISMA Statement. PLoS Med 6(6): e1000097. doi: 10.1371/journal.pmed1000097. For more information, visit www.prisma-statement.org.

Two investigators used EndnoteX7 software to conduct a preliminary assessment of the title and abstract of each document in the database based on the established criteria for inclusion in the study to select eligible studies. After a preliminary assessment, the full text of the selected literature would be evaluated, and the uncontrolled study, no randomization, inconsistent evaluation criteria, and similar data would be excluded. Any differences in screening that occurred during the screening study would be discussed in order to get consensus, if it still cannot be resolved, then the third author would be intervened. The primary selection process is shown in a PRISMA flow chart (Fig. 1)

#### 2.3.2 Data extraction and management

Two investigators independently extracted information from the included literature. The extracted content includes research design, random hiding and blinding, basic information of the included cases, intervention methods, observation indicators, and test results of the treatment group and the control group. The extracted literature data will be filled in a unified data statistics table. For studies that provide baseline and post-treatment data, we will estimate the change values by the method recommended by Cochrane. The complete PubMed search strategy is summarized in Table 1.

**Table 1:**
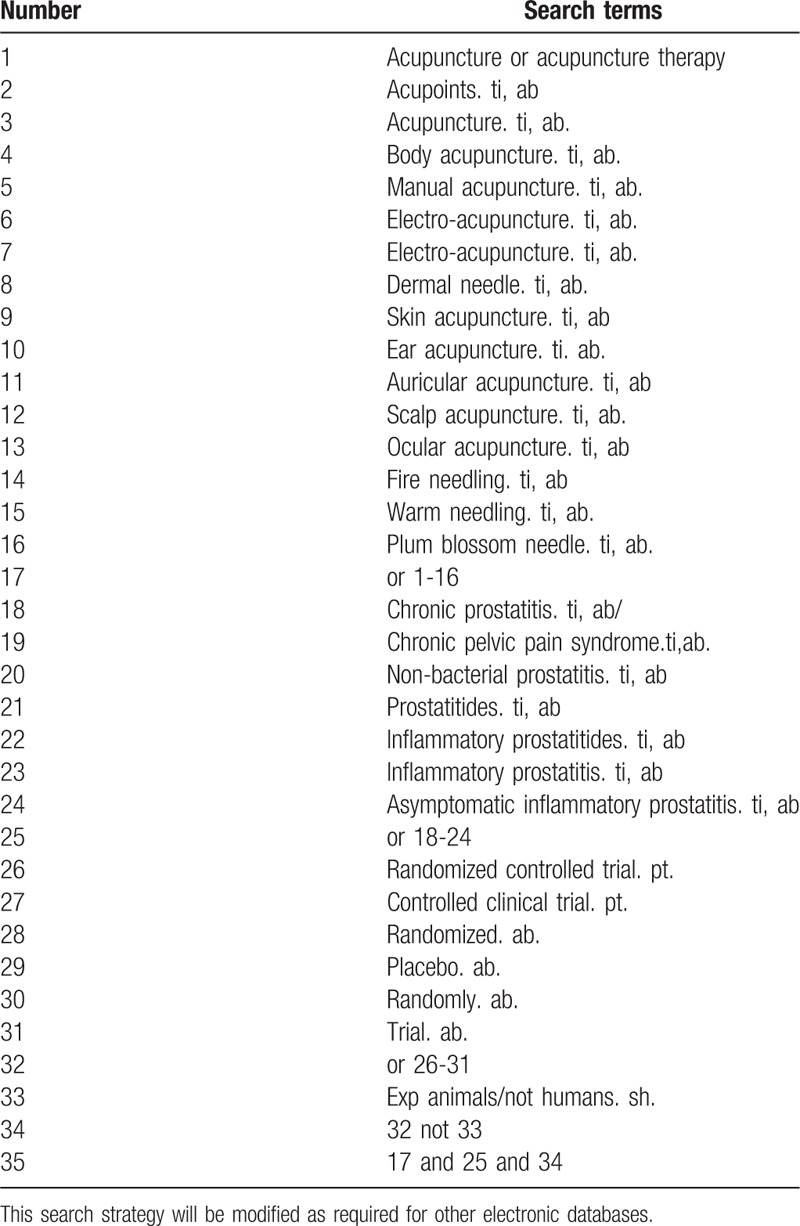
Search strategy used in PubMed database.

#### 2.3.3 Assessment of risk of bias in included studies

Two investigators will independently evaluate the methodological quality of the included literature by using the Cochrane Collaboration's ROB tool which includes whether the random method is correct, whether blinding is used, whether it is hidden, whether it is lost or quit, whether it uses intent-to-treat analysis, whether the data results are accurate, and other risks of bias. According to the relevant standards in the Cochrane Intervention System Evaluation Manual, it will be divided into low risk, high risk, and unclear.

#### 2.3.4 Dealing with missing data

In the event of data loss during the screening and extraction of literature data, first, we will actively investigate the cause of data loss. Then we will contact the experimental research author by telephone, mail, and so on to achieve the purpose of supplementing the missing data. If the lost data cannot be retrieved, we will only extract and analyze the useful data, besides, we will indicate the situation.

#### 2.3.5 Statistical analysis

##### 2.3.5.1 Pairwise meta-analysis

The numerical variable will be expressed as the normalized mean difference (standardized mean difference [SMD]) with a confidence interval (CI) of 95%. The heterogeneity of each pairwise comparison will be tested by chi-square test (test level α = 0.1). If there is no heterogeneity, a fixed effect model will be used. If there is significant heterogeneity between a group of studies, we will explore the reasons for the existence of heterogeneity from various aspects such as the characteristics of the subjects and the degree of variation of the interventions. Sensitivity analysis or meta-regression and subgroup analysis to explore possible sources of heterogeneity when necessary. We will use qualitative analysis of the funnel plot and graph symmetry to assess publication bias. Quantitative methods such as Begg testing and Egger testing will be used to help assess publication bias in the application.

##### 2.3.5.2 Network meta-analysis

We will use GeMTC 0.14.3 software to analyze the data. STATA 13.0 and WinBUGS 1.4.3 will be used to perform NMA to synthesize direct and indirect evidence. The NMA will mainly use the Bayesian Markov chain-Markov Chain Monte Carlo random effects model and simulate with 5 chains. The convergence of the simulation will be evaluated by using the potential reduction factor and the Gelman-ubin-rooks diagram. The choice of the final model will depend on the biased information standard (DIC) value. In general, models with smaller DIC values are better. The total effective rate is counted, and the odds ratio is used to analyze the statistic. The effect size is expressed in 95% CI, and the numerical variable is expressed as SMD. The treatment level for each result will operate on the cumulative sorting curve (SUCRA) interface. The evidence relationship incorporated into the study will be calculated by STATA. If there is a “closed loop,” the node splitting method will be used to evaluate the inconsistency of each loop.

#### 2.3.6 Assessment of heterogeneity

If there is significant heterogeneity between a group of studies, we will explore the reasons for the existence of heterogeneity from various aspects such as the characteristics of the subjects and the degree of variation of the interventions. Sensitivity analysis or subgroup analysis is performed as necessary to explain heterogeneity.

#### 2.3.7 Assessment of publication bias

The forest map and funnel plot were drawn and analyzed using Rev Man5.3 software, and the funnel plot was used to analyze potential publication bias.

#### 2.3.8 Grading the quality of evidence

The quality of evidence for the main outcomes will also be assessed with the grading of recommendations assessment, development, and evaluation approach. The evaluation included bias risk; heterogeneity; indirectness; imprecision; publication bias. And each level of evidence will be made “very low,” “low,” “moderate,” or “high” judgment.

## 3 Discussion

At present, there are vast of therapies in treating CP/CPPS; however, the efficacy is still unsatisfactory due to the particularity of the anatomical structure of prostate.^[21]^ Studies have shown that drug intervention can improve the overall NIH-CPSI score and ameliorate most of the symptoms of CP/CPPS patients to a certain extent, but there is no single drug can continue to significantly ameliorate all symptoms of CP/CPPS patients.^[22–23]^ TCM has a profound theoretical foundation and abundant clinical experience in the treatment of CP/CPPS.^[24]^ Acupuncture, 1 essential part of TCM, has the characteristics of few side effects, simple and easy to use, and has long been used to treat genitourinary diseases. Acupuncture therapy mainly achieves therapeutic effects by stimulating the body's righteousness and regulating the balance of qi and blood.^[25]^ In recent years, acupuncture therapy has been widely used in clinical trials of CP/CPPS. Recent studies have shown that acupuncture can alleviate the pain caused by CP/CPPS to a certain extent and improve the quality of lives of patients.^[26]^

Although abundant studies have evaluated the effectiveness of acupuncture in treating CP/CPPS, evaluation and comparison between various treatments are still insufficient. To the best of our knowledge, NMA has not been used in recent years to compare the effectiveness of acupuncture in the treatment of CP/CPPS. The results of NMA can provide a possible ranking for acupuncture treatment of CP/CPPS. We hope that the results will provide clinicians with the best options for treating CP/CPPS and provide them with research directions. Due to the limited number of relevant high-quality studies and the few sample size included, the strength of the arguments of the conclusions is to some degree limited. Therefore, we hope that more large-scale, high-quality randomized controlled trials should be necessary in the future. Besides, improving the quality of the original research and conducting high-quality multicenter RCTs to explore the clinical efficacy of acupuncture treatment of CP/CPPS is also indispensable, through which could make the conclusion more objective and reasonable.

## Author contributions

BW and HSL are the guarantor. JSW and BHB contributed to the conception of this review. JSW drafted the manuscript of the protocol and BHB revised it. HHD developed the search strategies while XDY and KGZ will implement them. JSW and BHB will also screen the potential studies, extract data and assess quality. In case of disagreement between the 2 data extractors, JSW will advise on the methodology and work as arbitrator. BHB will complete data synthesis. All authors approved the final version for the publication.

**Data curation:** Yi Lei, Xueyun He, Jingshang Wang.

**Formal analysis:** Xiaoyong Gong, Wei Zheng.

**Project administration:** Chaohui Xue.

**Supervision:** Jiajia Ma.

**Validation:** Jiajia Ma.

**Writing – original draft:** Yahui Xue, Yongqiang Li.

**Writing – review and editing:** Yi Lei, Bao Zhang.
